# Imaging Metabolically Active Fat: A Literature Review and Mechanistic Insights

**DOI:** 10.3390/ijms20215509

**Published:** 2019-11-05

**Authors:** Joseph Frankl, Amber Sherwood, Deborah J. Clegg, Philipp E. Scherer, Orhan K. Öz

**Affiliations:** 1 Department of Radiology, University of Texas Southwestern Medical Center, 5323 Harry Hines Blvd, Dallas, TX 75390-8542, USA; joseph.frankl@utsouthwestern.edu (J.F.); amber.sherwood@utsouthwestern.edu (A.S.); 2 College of Nursing and Health Professions, Drexel University, 10th Floor, Room 1092, 1601 Cherry Street, Mail Stop 10501, Philadelphia, PA 19102, USA; debclegg@outlook.com; 3 Department of Internal Medicine, Touchstone Diabetes Center, University of Texas Southwestern Medical Center, 5323 Harry Hines Blvd, Dallas, TX 75390-8542, USA; philipp.scherer@utsouthwestern.edu

**Keywords:** Brown adipose tissue, FDG, PET/CT, SPECT, fatty acids, acetate, carbon-13, MSOT, obesity

## Abstract

Currently, obesity is one of the leading causes death in the world. Shortly before 2000, researchers began describing metabolically active adipose tissue on cancer-surveillance ^18^F-fluorodeoxyglucose (FDG) positron emission tomography/computed tomography (PET/CT) in adult humans. This tissue generates heat through mitochondrial uncoupling and functions similar to classical brown and beige adipose tissue in mice. Despite extensive research, human brown/beige fat’s role in resistance to obesity in humans has not yet been fully delineated. FDG uptake is the de facto gold standard imaging technique when studying brown adipose tissue, although it has not been rigorously compared to other techniques. We, therefore, present a concise review of established and emerging methods to image brown adipose tissue activity in humans. Reviewed modalities include anatomic imaging with CT and magnetic resonance imaging (MRI); molecular imaging with FDG, fatty acids, and acetate; and emerging techniques. FDG-PET/CT is the most commonly used modality because of its widespread use in cancer imaging, but there are mechanistic reasons to believe other radiotracers may be more sensitive and accurate at detecting brown adipose tissue activity. Radiation-free modalities may help the longitudinal study of brown adipose tissue activity in the future.

## 1. Introduction

Obesity is caused by chronic excess of calories consumed relative to calories burned. Development of obesity is often accompanied by metabolic dysfunction including dyslipidemia and diabetes. Other associated diseases include cancer, cardiovascular disease, osteoarthritis, and nonalcoholic fatty liver disease [1]. Improving treatment of obesity and obesity-related conditions is a priority in the developed world. In the United States of America alone, obesity is estimated to contribute to over 175,000 excess deaths per year [2]. Healthcare costs attributable to obesity may now exceed $150 billion annually in the United States [3]. Major professional society guidelines recommend pharmacotherapy as a treatment option for obesity (body mass index [BMI] >30 kg/m^2^) and overweight with a BMI >27 kg/m^2^ and associated comorbidities such as type 2 diabetes, obstructive sleep apnea, or hypertension [4,5]. A recent systematic review found that currently approved medications have demonstrated efficacy between 40–75% for reducing weight by 5% over 52 weeks compared to 23% for placebo [6]. Some of the most efficacious medications are however also associated with treatment discontinuation because of adverse effects [6]. There remains an unmet need for efficacious and tolerable medications for the treatment of obesity and prevention of obesity-related complications.

**Figure 1:**
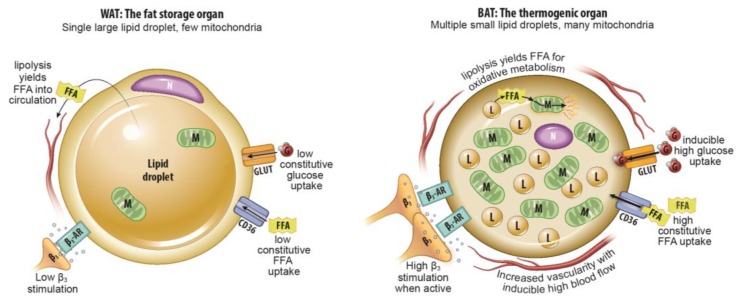
Summary of major physiological differences between white adipose tissue (WAT) and brown adipose tissue (BAT). A summary of major physiological differences relevant to imaging between WAT and BAT. FFA: free fatty acid, N: nucleus, M: mitochondria, β_3_-AR: beta-3 adrenergic receptor, G: glucose, L: lipid droplet, CD36: cluster of differentiation 36/fatty acid translocase, GLUT: glucose transporter.

One potential target for medical therapies aimed at reducing obesity and preventing associated metabolic dysfunction is metabolically active fat tissue. Human infants have classical interscapular brown adipose tissue that is of myogenic origin and is analogous to the primary thermogenic fat depots in mice and other small mammals [7]. This tissue is thought to recede quickly with age [7,8]. However, a significant proportion of human adults have metabolically active fat tissue in the supraclavicular and cervical areas as detected by ^18^F-fluorodeoxyglucose (FDG) positron emission tomography (PET) following cold exposure [9,10,11]. Unlike classical brown adipose tissue (BAT) with its relatively homogenous collection of multilocular adipocytes, metabolically active fat tissue in adults has multilocular adipocytes of adipogenic precursor origin interspersed among typical, unilocular white adipocytes [12]. Like adipocytes in classical BAT, multilocular adipocytes in this adult tissue (which goes by multiple terms including “beige” and “brite” adipose tissue, but will be called inducible BAT [iBAT] in this review, consistent with a National Institutes of Health [NIH] convened expert panel’s preferred terminology) generate heat and consume energy stores through mitochondrial uncoupling via Ucp1 [12,13]. Its presence is thought to decrease with age, obesity, and has been shown to be usually lower in men than women in several studies [14,15,16], although in clinical practice there is wide variation. Figure 1 summarizes the physiological differences between thermogenic BAT and lipid-storing WAT.

Most analyses of inducible BAT in human adults have used FDG-PET. However, this modality has several limitations for the purpose of identifying and quantifying iBAT: it does not detect inactive iBAT as judged by small animal studies assessed by other imaging modalities. Furthermore, it relies on glucose uptake, whereas iBAT generates most of its energy from stored triglycerides. Also, FDG-PET criteria for identifying iBAT vary between studies [13,17,18]. Molecular imaging strategies that probe oxidative metabolism or free fatty acid uptake may therefore be more sensitive to iBAT presence and activity than FDG-PET. Application of any radiotracer-based molecular imaging study in longitudinal studies with human subjects must take into account radiation dose. Magnetic resonance imaging (MRI) allows for repeated scans without radiation and can take advantage of the known difference in typical fat fraction and mitochondrial density between iBAT and white adipose tissue (WAT) [19]. However, intrasubject variability and iBAT cellular composition changes over time may limit the utility of current MRI methods [20,21]. Emerging techniques with multispectral optoacoustic tomography and ^13^C-MRI may eventually be used more often in research of iBAT. However, the optimal imaging strategy for detection and longitudinal study of iBAT in humans remains to be determined. In this review, we describe different techniques, their application, limitations, and areas for further research.

## 2. Anatomic Imaging

### 2.1. Computed Tomography

**Figure 2:**
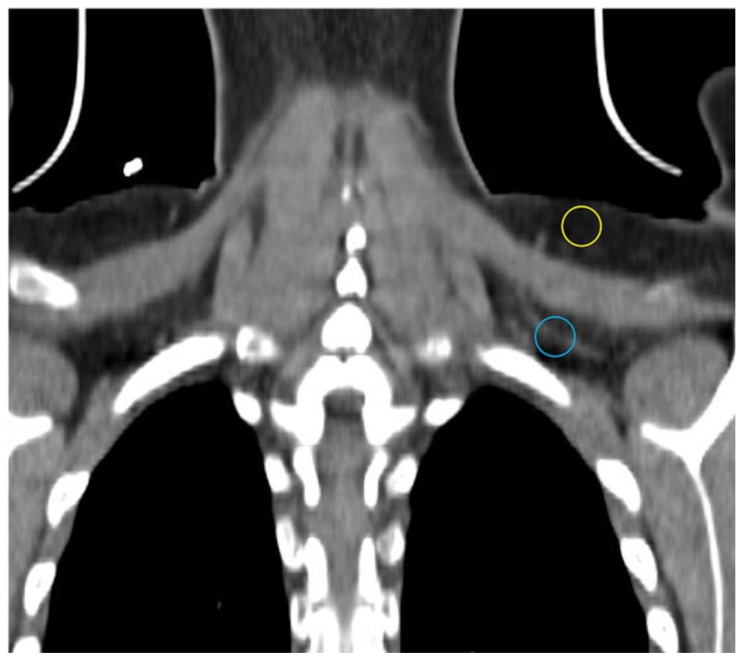
CT showing different density and vascularity of BAT versus WAT. Subcutaneous WAT with a darker appearance on conventional display and fewer vessels (yellow circle) compared with BAT (blue circle).

Computed tomography (CT) utilizes x-rays to generate a 3-dimensional dataset of tissue attenuation values that can be reconstructed into images in multiple planes. Different x-ray attenuation values of tissues form the basis of CT contrast. CT images have high resolution. iBAT is most often encountered in humans on CTs obtained during PET-CT studies in cancer patients. Although CT does not directly provide any functional data, radiodensity, which in the case of adipose tissue is largely determined by the ratio of fat to water, can be compared between different locations and quantitated using the Hounsfield scale [22,23,24]. The multilocular Ucp1-rich adipocytes have a higher fraction of water than their WAT counterparts and thus should be more radiodense [22,25]. Indeed, Prodhomme and colleagues found adipose tissue identified as metabolically active with FDG-PET has higher mean Hounsfield units (HU) than FDG non-avid WAT (−32.6 ± 26 vs. −99.6 ± 18.8 HU) [16]. HUs increase further with activation and utilization of lipid by BAT in rodents and humans [26,27,28]. Obese individuals have lower mean Hus in typical location of iBAT as in the supraclavicular region consistent with greater lipid content, and mean HU increases with weight loss [29]. In addition to supraclavicular depots, relatively high-attenuation fat depots in cervical, axillary, mediastinal, and intraabdominal locations may be iBAT [10,30,31]. CT demonstrates the known greater vascular density in BAT compared to WAT in our experience (Figure 2) [32].

### 2.2. Magnetic Resonance Imaging

Conventional proton MRI takes advantage of the small magnetic field produced by hydrogen atoms. Broadly, the MRI scanner produces a strong magnetic field that causes hydrogen atoms to align; so there is a small net magnetization aligned with the external magnetic field. Radiofrequency pulses flip hydrogen atoms out of alignment. The rate at which hydrogen atoms return to alignment with the external magnetic field is termed T1. When the aligned magnetic fields of hydrogen atoms transverse the external magnetic field, the rate at which they leave alignment with the transverse plane is termed T2. T1- and T2-weighted images are the most common MR images [33]. Additional MR acquisitions and image processing techniques can produce images that reflect the fat/water ratio and mitochondrial density, which both may be helpful in BAT research [19].

**Figure 3:**
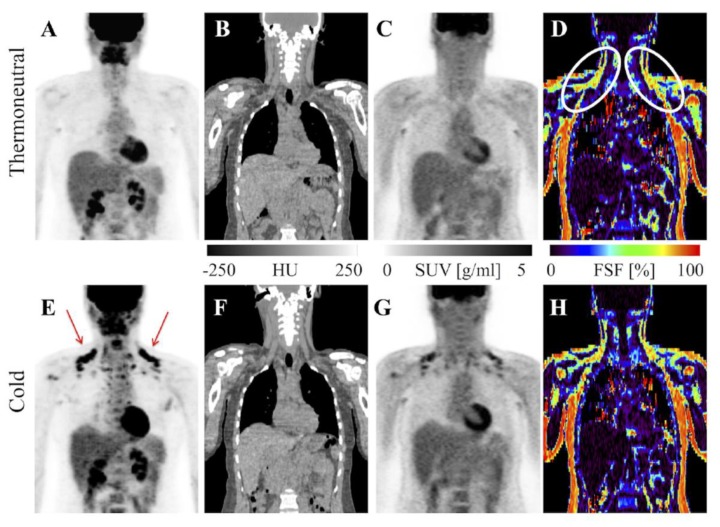
MRI vs. FDG-PET/CT in detection of BAT. FDG-PET/CT and MRI of a healthy BAT-positive research subject. FDG uptake is visible in supraclavicular BAT with cold exposure on the PET maximum intensity projection (MIP; **E**, red arrows) and absent from the thermoneutral PET MIP (**A**). CT shows adipose tissue in the same region (**B**,**F**) and PET shows increased standardized uptake value there after exposure to cold (**C**,**G**). MRI-derived fat signal fraction from the same subject shows adipose tissue with relatively low levels of fat (white ovals) in the same region at thermoneutral and cold conditions (**D**,**H**). Image reproduced with permission from reference 41.

Stojanovska and colleagues showed water-fat imaging distinguishes ex vivo samples of BAT, WAT, and mixed BAT/WAT from each other. Specifically, the proton density fat fraction as a percent was significantly lower in BAT compared to other samples [34]. As mentioned above, this is because BAT has less lipid deposits than WAT. A similar technique successfully discriminated BAT from WAT in rodents and additionally correlated the two different depots with specific CT HU ranges [35]. Dixon-based MRI, a technique for generating fat and water images, and several investigational water-fat algorithms have differentiated iBAT from WAT in human subjects [19,36,37,38,39]. Results were confirmed by FDG uptake detected separately on PET/CT or in the same scan via PET/MRI [19,38,40]. At thermoneutral conditions, the MR fat fraction differentiates iBAT from WAT with greater sensitivity than FDG-PET/CT (Figure 3) [19,41,42]. Cold stimulation accentuates the fat-fraction difference between BAT and WAT, as even short-duration cold exposure induces lipid utilization by Ucp1-rich adipocytes [20,39,40]. A limitation of fat-fraction MR in this setting is variability between subjects in the optimal cutoff value to distinguish BAT from WAT [21]. Romu and colleagues showed calibrating fat images based on the T2*-derived fat content of WAT to form relative fat content images can identify BAT in rats and may avoid the pitfall of inter-subject variability by using an internal control [35].

R2* and T2* images also differentiate iBAT from WAT. T2* is a time constant generated from the signal decay immediately following the initial excitation pulse and R2* is the inverse of T2* [43]. These parameters are influenced by the iron content of mitochondria, which are present at high density in iBAT [44,45]. Greater blood flow to BAT/iBAT compared to WAT may also contribute to R2* and T2* differentiation [41]. Lundström and colleagues showed R2* maps have a high degree of overlap with MR fat fraction maps of iBAT [37]. However, unlike fat-fraction, R2* does not change significantly with acute cold exposure likely because mitochondrial density does not change [41].

**Figure 4:**
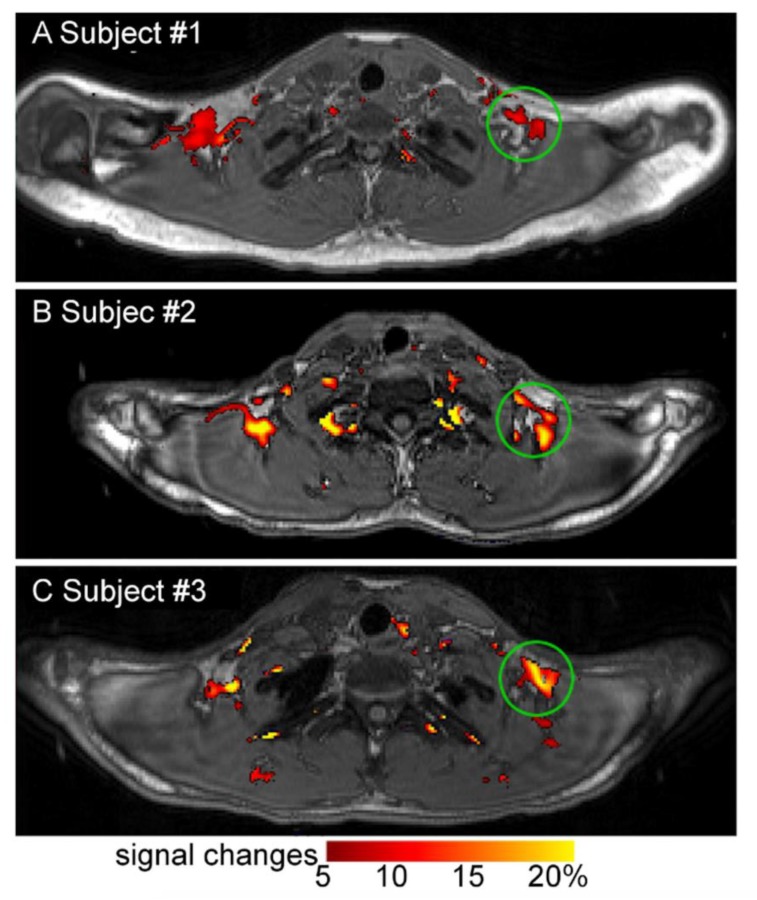
Change in blood oxygen level dependent MRI signal in human BAT with cold exposure. Change in blood oxygen level dependent MRI signal in supraclavicular BAT with cold exposure (13–16 °C) in healthy human research subjects. Images **A**, **B**, and **C** are representative images of 3 subjects. Image reproduced with permission from reference 47.

There are several emerging MR-based techniques. Functional MRI may be applied to detect changes in blood flow in activated iBAT in future research although few investigators have used it in BAT research thus far [46]. Blood oxygenation level dependent functional MRI signal increased in iBAT upon cold exposure in one small (*n* = 3) study with adult humans (Figure 4) [47]. MR thermometry is an emerging technique that measures tissue temperature and may provide an additional measure of iBAT activity [40].

## 3. Molecular Imaging with Radiotracers

### 3.1. ^18^F-Fluorodeoxyglucose-PET

FDG-PET/CT is the de facto gold standard imaging study in BAT research [13]. However, a mechanistic understanding of FDG uptake and BAT physiology challenges FDG-PET/CT’s current role. FDG is transported into cells via glucose transporters. It is phosphorylated, like glucose, to FDG-6-phosphate, but does not undergo further metabolism [48]. Trapped FDG-6-phosphate is detected in locations with greater glucose uptake and utilization. BAT’s primary source of energy is the intracellular triglyceride pool [49]. FDG uptake occurs as metabolically active adipocytes replenish their lipid pools, but this is a downstream event from BAT thermogenesis. Some amount of glucose uptake also occurs via acute β_3_-adrenergic receptor stimulation [50]. Nevertheless, FDG-PET/CT is an important technique in BAT research from a historical perspective and remains the most widely used imaging study in this setting. Large retrospective reviews of atypical supraclavicular FDG uptake in oncology patients were among the first indication of iBAT prevalence in adult humans [51,52]. Dedicated BAT research protocols have since used FDG-PET/CT to establish much of the current BAT literature. Advantages of FDG include its availability and the high number of clinical scans performed at large oncology centers, which generate databases amenable to BAT research.

**Figure 5:**
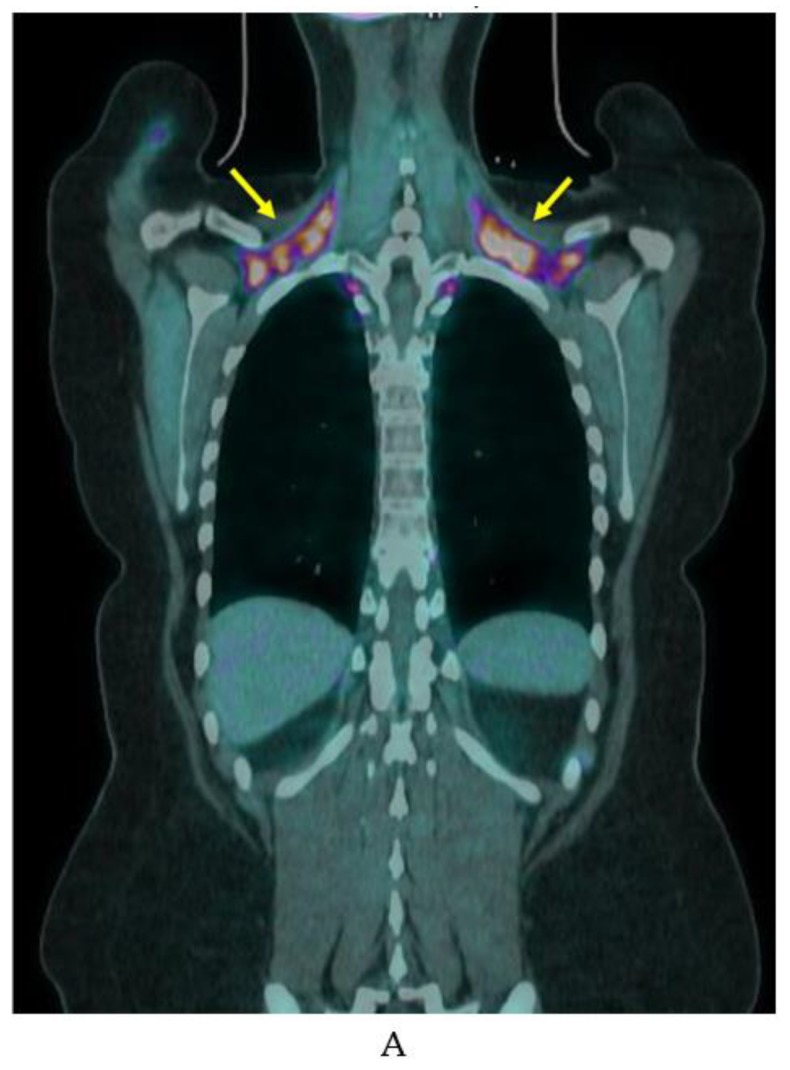

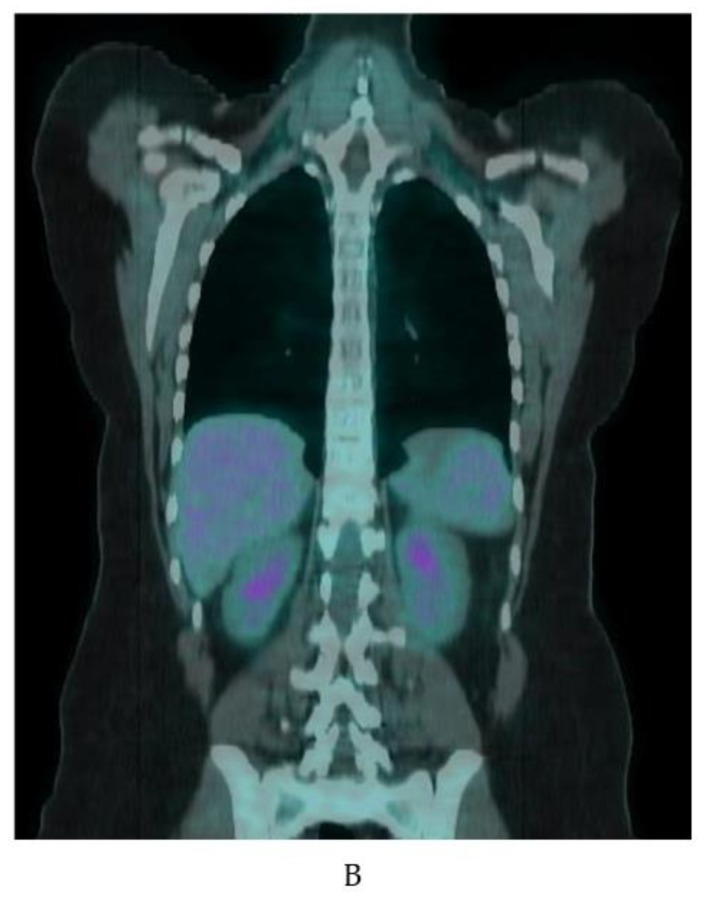
Example of variable iBAT uptake on FDG-PET/CT. FDG-PET/CT in a woman in her 20s showing marked BAT uptake in (**A**) November (yellow arrows) and no detectable BAT FDG uptake on a scan (**B**) 6 months later.

The rate of iBAT detected on a single FDG-PET/CT scan in large oncology cohorts is between 5–10% [14,16,53]. However, rate of detection rises substantially with repeated scanning or cold stimulation, although estimated prevalence varies widely between 20–100% [53,54,55]. Figure 5 shows an example of a patient with variably detectable iBAT. Indeed, seasonal changes in temperature impact iBAT prevalence estimates, limiting the utility of retrospective cohort studies [31]. Female gender and younger age have been associated with increased iBAT prevalence in large retrospective studies [15,56]. Lower adiposity, particularly visceral adiposity, is also associated with increased iBAT detection on FDG-PET/CT [15,57]. However, the lack of sensitivity to inactive BAT presents a problem to clinical BAT research as the most relevant subjects are obese and metabolically unhealthy.

**Figure 6:**
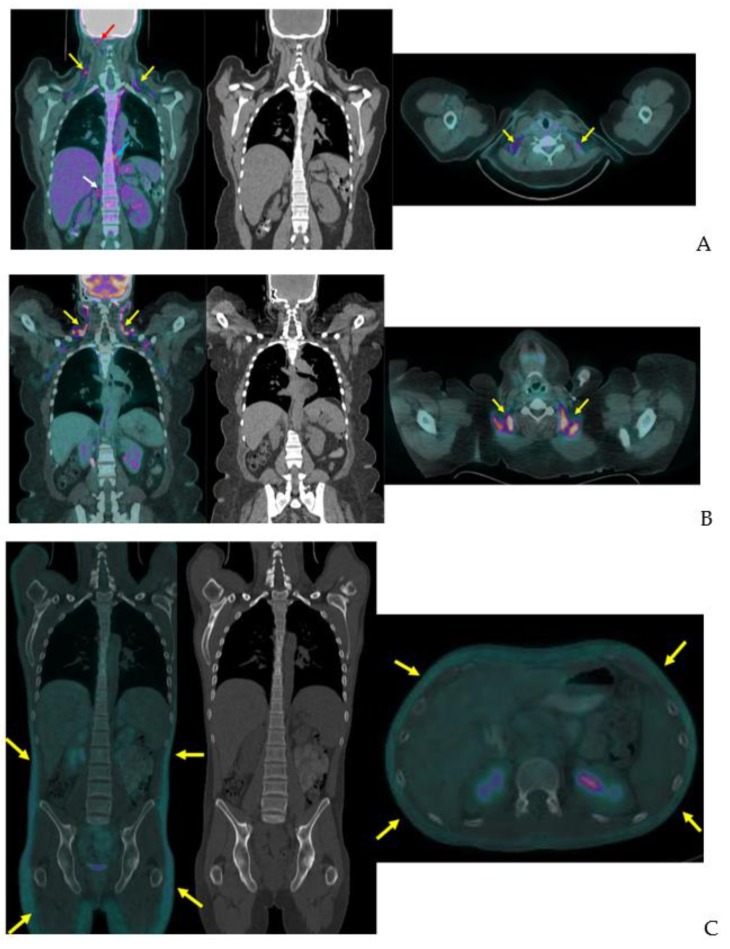

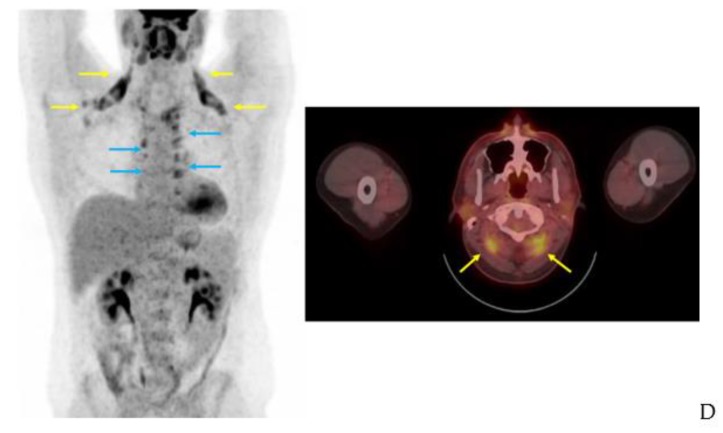
Variation of BAT FDG uptake in clinical patients. Fused coronal FDG-PET/CT, coronal CT, and fused axial FDG-PET/CT (**A**–**C**). (**A**) BAT FDG uptake in a woman with normal BMI. Note supraclavicular (yellow arrows), skull base (red arrow), paraesophageal (blue arrow), and perinephric (white arrow) uptake. (**B**) Supraclavicular BAT in an obese woman (yellow arrows). (**C**) Diffuse FDG uptake in subcutaneous WAT in a male patient (yellow arrows). (**D**) A male patient with extensive supraclavicular FDG uptake extending into the upper neck (yellow arrows) and paravertebral BAT activity (blue arrows).

Prospective studies often use cooling protocols to induce iBAT activation. Leitner and colleagues showed a 20 min cold exposure is sufficient to activate iBAT in healthy adult volunteers [58]. In their study, 3 h of rewarming after cold exposure decreased FDG uptake by iBAT to near baseline [58]. Indeed, iBAT is so temperature sensitive a NIH expert panel recommends reporting outdoor temperature, season, and geographic location in any FDG-PET/CT study of iBAT [13]. Despite the strong association with cooler temperatures, we routinely see active iBAT on warm days in our large oncology practice where the uptake room is held at a constant temperature warm enough to prevent shivering. Figure 6 shows examples of the wide variation of uptake in clinical patients.

The above-mentioned NIH panel released the first guidelines on reporting FDG-PET findings in BAT research, named the Brown Adipose Reporting Criteria in Imaging Studies (BARCIST 1.0). Prior to that, criteria to identify metabolically active fat tissue varied greatly [18]. Their recommendations were to label regions with HUs between −190 and −10 and standardized uptake values (SUV) of 1.2/(lean body mass)/(body mass) as brown fat. Additional recommendations include reporting subject body weight, height, and BMI; reporting liver and descending aorta SUV to establish background activity; having subjects fast 6 h prior, refrain from eating fatty meals 24 h prior, and not consume caffeine or capsinoids within 48 h of scanning; and excluding smokers and individuals on sympathomimetics and sympatholytics [13].

### 3.2. ^18^F-Fluoro-6-Thia-Heptadecanoic Acid (FTHA) PET

**Figure 7:**
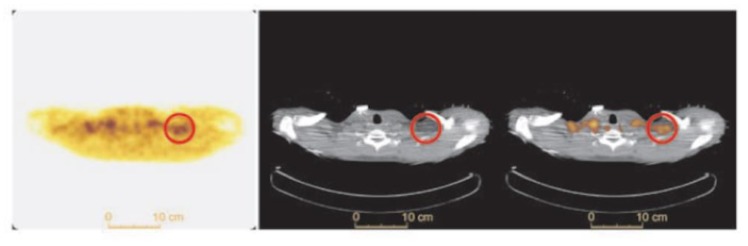
BAT FTHA uptake in a healthy human subject. Transverse FTHA-PET (left panel), CT (middle panel) and fused PET/CT (right panel) in a healthy subject after 1 h of cold exposure. Supraclavicular BAT is shown in the red circle. Image reproduced with permission from reference 28.

FTHA is a fatty acid probe that is transported into cells by fatty-acid transport protein gets trapped in the mitochondria after commitment to the mitochondrial fatty acid oxidation pathway [59]. It was initially developed to assess oxygenation of myocardium [59]. It has since been used by several groups to assess fatty acid uptake by BAT (Figure 7). Labbé and colleagues showed FTHA localizes to BAT as FDG does with cold stimulation in rodents [49,60]. FTHA actually identified additional periaortic and cervical BAT depots FDG did not manage to pinpoint [49]. Ouellet showed dynamic FTHA uptake over 30 min increases with in adult human iBAT with cold exposure using Patlak graphical analysis [28]. Dadson and colleagues found dynamic FTHA uptake over 15 min increased in iBAT after bariatric surgery in a cohort of morbidly obese women using graphical analysis with concomitant increase in CT radiodensity, indicating lower intracellular iBAT lipid stores [29].

### 3.3. ^123^I-Beta-Methyl-Iodophenyl-Pentadecanoic Acid (BMIPP) Single-Photon Emission Computed Tomography (SPECT)/CT

**Figure 8:**
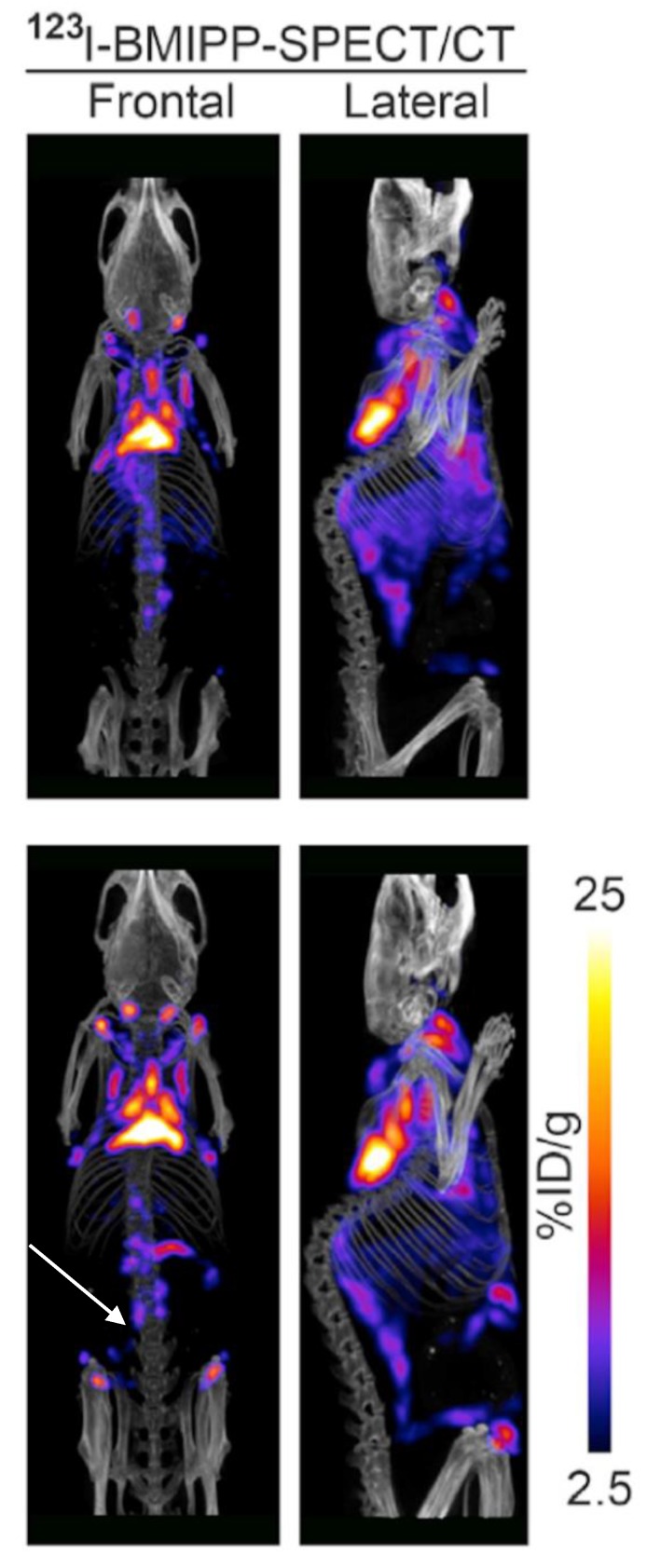
BMIPP-SPECT/CT with and without beta-adrenergic stimulation. BMIPP-SPECT/CT scanning for the patterns of fatty acid uptake in the metabolic active fat tissues of adult mice at room temperature after 7 days of vehicle treatment (top) or 7 days of treatment with the β_3_-adrenergic agonist CL-316243 (bottom row). Treatment increased uptake in classical interscapular BAT cervical depots, and inducible anterior abdominal subcutaneous (arrow) and inguinal metabolically active fat tissue. Image reproduced with permission from reference 30.

BMIPP is a fatty acid tracer routinely used for cardiac SPECT/CT in Japan [61]. It is transported into the cell by CD36 and trapped during incomplete β oxidation [62,63]. Although developed in the United States, it is not approved for clinical use by the Food and Drug Administration. Research with BMIPP likely requires on-site synthesis. However, synthesis does not require a cyclotron as the long (13 h) half-life of ^123^I makes it suitable for transport [64]. Additionally, ^125^I-labeled BMIPP produces high-resolution images on preclinical scanners and has a half-life of nearly 60 days [65]. In our hands, BMIPP identified BAT depots missed by FDG-PET/CT in mice (Figure 8) [30]. Additionally, BMIPP-SPECT/CT has a much more favorable signal-to-noise compared to FTHA-PET/CT [30]. We believe it is a measure of flux through the triglyceride pool.

### 3.4. ^11^C-Acetate-PET

Acetate is transported into cells via monocarboxylate transporters and converted to acetyl-CoA by acyl-CoA synthetases prior to catabolism in the citric acid cycle or use in biosynthetic reactions [66,67]. Clinically, ^11^C-acetate is used to measure myocardial oxygen consumption and malignant lipogenesis by slow-growing, FDG non-avid tumors [68,69,70,71,72]. A fitted monoexponential function (K_mono_) of clearance beginning after blood pool stabilization is used in cardiac studies to estimate oxidative metabolism via the citric acid cycle [69,70]. ^11^C-acetate’s use in cancer imaging is similar to FDG-PET with SUV_max_ and SUV_mean_ more often followed [71,72]. These values reflect incorporation of the radiotracer into fatty acids. Wider use is limited by the need for an on-site cyclotron because of carbon-11’s short half-life (~20.38 min) [73].

^11^C-acetate’s ability to assess oxidative metabolism is utilized in study of metabolically active fat tissue in rodents and humans [27,28,60,74]. Clearance after blood pool stabilization at 2–4 min through up to approximately 17.5 min after radiotracer injection is due to conversion into CO_2_ in the citric acid cycle [68]. Acute and chronic cold exposure increase ^11^C-acetate K_mono_ by classical BAT depots in rodents and iBAT in adult humans [27,28,49,60]. Nicotinic acid, a lipolysis inhibitor, substantially reverses this effect, indicating stored intracellular triglycerides provide much of the energy for oxidative metabolism in these tissues [49]. More recent studies have used a 3 compartment kinetic model with ^11^C-acetate K_2_ estimating CO_2_ production from the citric acid cycle [49,60], which may provide a better assessment of iBAT metabolic activity than other methods because it is sensitive to mobilization of intracellular energy stores. However, neither ^11^C-acetate K_mono_ or K_2_ have been compared to direct measurement of oxidative activity in adipose tissue.

### 3.5. Other Radiotracers

**Figure 9:**
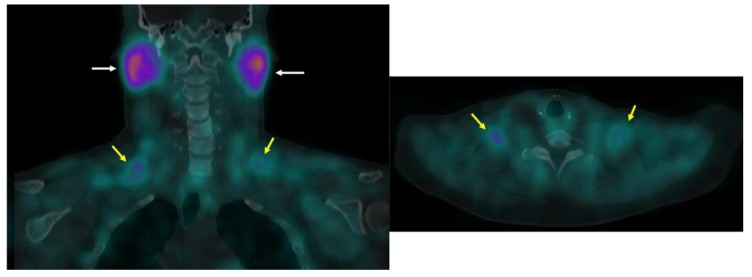
^99m^Tc-MIBI uptake in iBAT on a clinical scan. Supraclavicular BAT uptake shown coronal and axial fused ^99m^Tc-MIBI-SPECT/CT (yellow arrows). Salivary gland uptake (white arrows) is normal.

BAT is detected with other clinical and investigational radiotracers. ^123^I-metaiodobenzylguanidine (MIBG) SPECT/CT localizes sympathetic innervation by using a labeled norepinephrine analogue [75]. MIBG localizes BAT with similar sensitivity to FDG-PET/CT but is less temperature dependent [9,75,76]. The ^11^C PET tracer and norepinephrine analog ^11^C-meta-hydroxyephedrin has demonstrated sensitivity to BAT at thermoneutral conditions and its activity correlates with FDG uptake with acute cold in humans [77]. The commonly used SPECT/CT tracer ^99m^Tc-methoxyisobutylisonitrile (MIBI), which localizes to tissues with high mitochondrial density, detects BAT in a non-temperature dependent manner as shown in an example from our clinical practice (Figure 9) [76,78]. ^15^O-labeled water PET/CT has been used in this setting to measure blood flow, which provides data to estimate tissue-specific metabolic rate using arterial oxygenation and known oxygen extraction fractions [77]. Din and colleagues combined ^15^O-labeled water PET/CT with inhaled ^15^O administration to directly measure tissue metabolic rate in humans [79]. The short (2 min) half-life of ^15^O allows for repeat PET scanning with another tracer on the same day [77,79]. Investigational radiotracer currently include the mitochondrial outer membrane translocator protein, visualized with ^18^F-FEPPA PET/CT and ^11^C-PBR28 PET/CT, the cannabinoid type 1 receptor, visualized with ^18^F-FMPEP-d2 PET/CT [80,81,82,83], and PD-L1, visualized with radiolabeled antibodies [84,85].

## 4. Emerging Technologies

BAT research invites application of novel imaging techniques. Inconsistent results from FDG-PET/CT studies limit our understanding of the tissue’s relevance in human physiology and disease [86]. We briefly review 4 techniques that may assist future BAT research: xenon-enhanced imaging, hyperpolarized C-13 MRI, contrast-enhanced ultrasound, and optoacoustic imaging.

Xenon is a highly lipophilic gas transported in blood after inhalation. As BAT is more vascularized than WAT, xenon preferentially accumulates in BAT [87,88]. Branca and colleagues first utilized xenon as a substrate for hyperpolarized MRI, finding it was more sensitive to BAT presence in obese mice than FDG-PET/CT or MRI fat-fraction [87]. Next, they showed xenon-enhanced CT was more sensitive than FDG-PET/CT for BAT in mice and rhesus monkeys, and highly specific [88]. The authors importantly note FDG-PET/CT is most limited in obese subjects, the population of greatest interest in BAT research.

**Figure 10:**
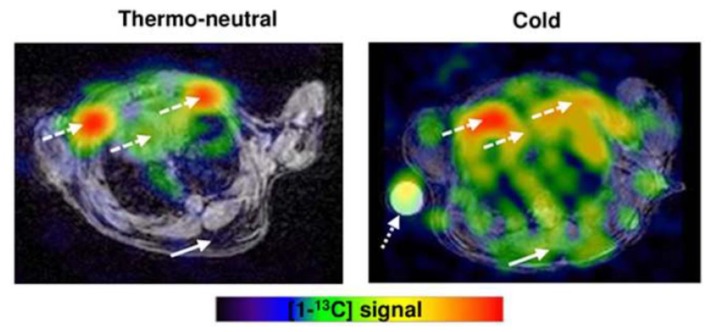
Hyperpolarized ^13^C-pyruvate MRI with cold exposure in a mouse. Hyperpolarized ^13^C-pyruvate MRI shows increased BAT activity in a mouse after cold exposure (solid arrow). The dashed arrows show the heart and large vessels and dotted arrow is a phantom. Image reproduced with permission from reference 92.

Carbon-13 produces a small magnetic field like hydrogen that can be utilized in MRI. However, its low isotopic abundance limits imaging without enrichment. Dissolution dynamic nuclear polarization (DNP) increases polarization of nuclear spins, greatly enhancing the generated magnetic field [89]. Commercial DNP systems now produce hyperpolarized ^13^C agents, most often pyruvate, for imaging studies. MRI obtained within about 1 min of hyperpolarized ^13^C-pyruvate injection show signal from downstream intermediates, notably lactate and bicarbonate, that indicate the rate of anaerobic (lactate) and aerobic (bicarbonate) metabolism in tissues [90]. The ratio of lactate/bicarbonate has been proposed as likely the most accurate metric generated in hyperpolarized ^13^C-pyruvate experiments as absolute values of ^13^C-lactate and ^13^C-bicarbonate are susceptible to effects of probe delivery and its membrane transport [90]. In a study by Lau and colleagues, hyperpolarized ^13^C-pyruvate MRI detected increased ^13^C-bicarbonate and ^13^C-lactate in BAT-associated interscapular regions of mice following norepinephrine stimulation [91]. Riis-Vestergaard then showed hyperpolarized ^13^C-pyruvate MRI of mice localizes BAT to the same regions as FDG-PET/MRI. The magnitude of increased metabolized pyruvate is similar to the magnitude of increased FDG uptake following cold stimulation, and there is increased anaerobic metabolism in BAT following cold stimulation (Figure 10) [92]. They also showed BAT increases metabolic activity independent of increased blood flow by adjusting for blood flow [92]. Advantages of this technique include lack of radiation and the ability to detect metabolic activity independent of blood flow. Disadvantages include limited availability, insensitivity to inactive BAT, and reliance on an upstream metabolic process.

Contrast-enhanced ultrasound (CEUS) with microbubbles estimates blood flow without radiation. It was first shown to detect BAT activation, as proven by histology, in mice [93]. Next, Flynn and colleagues showed it can detect increased blood flow with cooling in iBAT in human subjects with FDG-avid iBAT deposits [94]. CEUS application is limited by its reliance on iBAT activation and superficial field of view. However, the most important metabolically active fat pads in humans are within CEUS’s field of view and FDG-PET/CT is also limited by a reliance on iBAT activation. The major advantages of CEUS are its low cost and lack of radiation.

**Figure 11:**
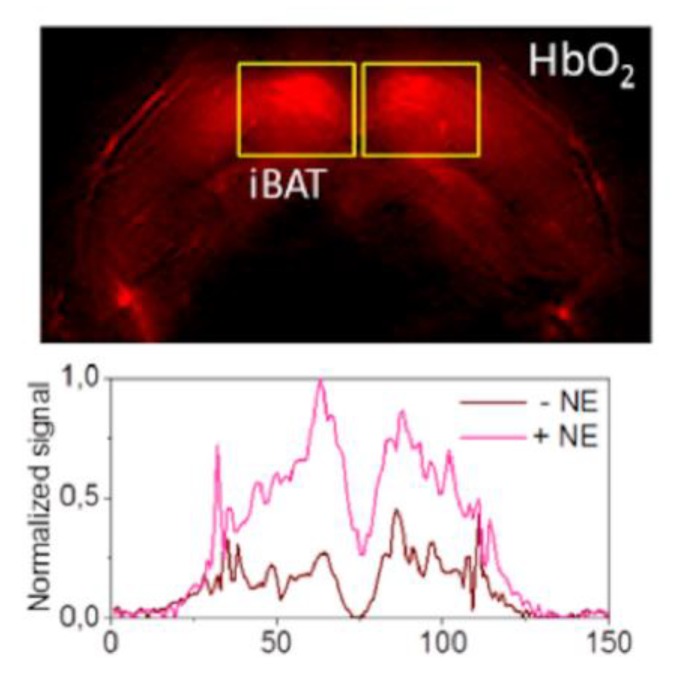
MSOT HbO_2_ map after norepinephrine treatment. MSOT image unmixed for HbO_2_ signal of murine interscapular BAT after treatment with norepinephrine (NE; top panel). BAT pads are shown in rectangles. The bottom panel shows the horizontal intensity profile with and without NE treatment. Image reproduced with permission from reference 95.

Multispectral optoacoustic tomography (MSOT) operates by pulsing light at a range of wavelengths for ultrashort periods of time triggering thermoelastic absorption by photoabsorbing molecules, which generates detectable mechanical waves at ultrasonic frequencies. MSOT arrays detect waves that can be reconstructed into images by unmixing known photoabsorption spectra of photoabsorbing biological molecules [95]. The Ntziachristos group recently showed MSOT can differentiate BAT from WAT in vivo and detect BAT activation by measuring real-time change in hemoglobin gradients in mice with a commercially available device (Figure 11) and in humans with a laboratory-modified handheld device [17]. Although wider use is limited by availability, the technology has the promise of a radiation-free, fast, and inexpensive means for repeatedly measuring iBAT activity in humans.

## 5. Summary

**Figure 12:**
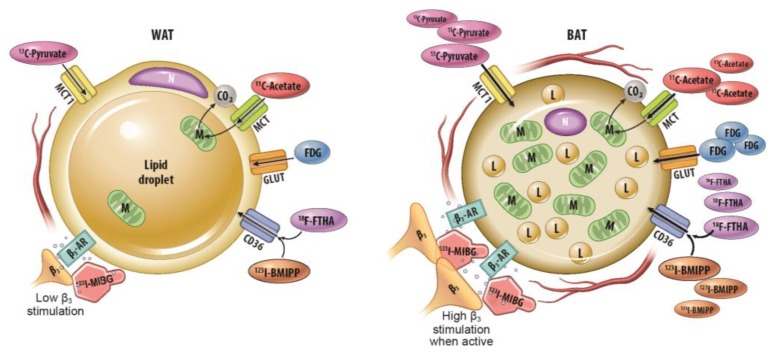
Physiological underpinnings of key imaging modalities for BAT research. The physiological basis for key imaging modalities in brown adipose tissue (BAT) research includes changes in inner action, vascularity, fat fraction, mitochondrial activity and key substrate transport and utilization. Comparison is made to white adipose tissue (WAT). N: nucleus, M: mitochondria, L: lipid droplet, FDG: ^18^F-Fluorodeoxyglucose, FTHA: ^18^F-Fluoro-6-Thia-Heptadecanoic Acid, BMIPP: ^123^I-Beta-Methyl-Iodophenyl-Pentadecanoic Acid.

FDG-PET/CT is the de facto gold standard imaging study in BAT research. The relatively frequent presence of metabolically active fat depots in adult humans was first noted and the largest human studies in this field were gleaned from review of FDG-PET/CTs obtained for cancer surveillance and staging [51,52,56,96]. However, it is not prone to visualization of inactive BAT, probes a molecular event (glucose transport) upstream of BAT activity that is influenced by numerous other factors, it is expensive, and it involves radiation. However, advances in hardware and research protocols may allow for substantial radiation reduction in the near future [97]. Molecular imaging with other agents may improve sensitivity and specificity. Fatty acid probes, including ^18^F-FTHA and ^123^I-BMIPP, may detect BAT depots missed by FDG [30,49]. Dynamic imaging with ^11^C-acetate provides information about oxidative metabolism and may therefore yield the useful data about BAT activity among molecular probes. Xenon-enhanced imaging appears may improve sensitivity in obese subjects [87,88]. Further advances in hyperpolarized MRI may allow more comprehensive evaluation of in vivo metabolism. Promising for repeated imaging of human subjects, contrast-enhanced ultrasound and multispectral optoacoustic tomography can detect iBAT activation without radiation [17,95]. Multispectral optoacoustic tomography can also differentiate BAT from WAT in the unstimulated state [17]. The physiological basis of key imaging modalities is summarized in Figure 12 and Table 1.

## 6. Conclusions

An ideal imaging study for human BAT research could detect BAT both when it is undergoing uncoupled mitochondrial oxidation and taking up fuel from the bloodstream and when it is not of high sensitivity. It could be repeated with limited or no radiation. Current methods typically require BAT induction with cooling to reach high levels of sensitivity. A reasonable humans-subjects research protocol for a BAT-activating intervention with current technologies is a screening FDG-PET/CT following a cooling protocol with a baseline radiation-free assessment (such as CEUS or MSOT where available) of identified depots’ metabolic activity followed by sequential assessment of BAT activity. Further research may allow screening with more promising molecular studies with ^11^C-acetate or fatty acid tracers without the need for validation with FDG-PET/CT.

## Figures and Tables

**Table 1:** Major differences between WAT and BAT on key imaging modalities. Comparison of WAT and BAT on key imaging modalities in BAT research. The relative finding of key factors are listed.

Modality	Feature	WAT Finding	BAT Finding	Radiation	References
CT	Density (HU)	↓	↑	+	16, 22, 25–28
MRI	Fat fraction	↓	↑	-	34, 36–39
MRI	Mitochondrial density (T2*/R2*)	↑	↓	-	37, 43, 44
FDG-PET/CT	SUV	↓	↑	++	13, 52–57
FTHA-PET/CT	Fractional uptake rate	↓	↑	++	28, 48, 59
BMIPP-SPECT/CT	Uptake	↓	↑	++	30
^11^C-Acetate-PET/CT	K_mono_	↓	↑	++	27, 28, 48, 59
^11^C-Acetate-PET/CT	K_2_	↓	↑	++	48, 59
Hyperpolarized ^13^C MRI	Total signal	↓	↑	-	90, 91
Contrast-Enhanced Ultrasound	Blood flow	↓	↑	-	92-93

↓: decreased, ↑: increased, -: no ionizing radiation, +: ionizing radiation present, ++: more ionizing radiation present.

## Abbreviations

BMI	Body mass index
PET	Positron emission tomography
BAT	Brown adipose tissue
iBAT	Inducible brown adipose tissue
MRI	Magnetic resonance imaging
WAT	White adipose tissue
CT	Computed tomography
HU	Hounsfield unit
NIH	National Institutes of Health
BARCIST	Brown Adipose Reporting Criteria in Imaging Studies
SUV	Standardized uptake values
FTHA	Fluoro-6-thia-heptadecanoic acid
BMIPP	Beta-methyl-iodophenyl-pentadecanoic acid
SPECT	Single-photon emission computed tomography
MIBG	Metaiodobenzylguanidine
MIBI	Methoxyisobutylisonitrile
DNP	Dissolution dynamic nuclear polarization
CEUS	Contrast-enhanced ultrasound
MSOT	Multispectral optoacoustic tomography
